# Development and Pilot Testing of a Booklet Concerning Medications That Can Increase the Risk of Falls in Older People

**DOI:** 10.3390/ijerph20010404

**Published:** 2022-12-27

**Authors:** Mohammad Suhaidi Shaari, Mohd Shahezwan Abd Wahab, Izzati Abdul Halim Zaki, Rosmaliah Alias, Muhammad Harith Zulkifli, Aida Azlina Ali, Nur Wahida Zulkifli, Farhana Fakhira Ismail, Mizaton Hazizul Hasan, Rulia Meilina, Long Chiau Ming, Ching Siang Tan

**Affiliations:** 1 Faculty of Pharmacy, Universiti Teknologi MARA (UiTM) Cawangan Selangor, Kampus Puncak Alam, Puncak Alam 42300, Malaysia; 2 Non-Destructive Biomedical and Pharmaceutical Research Centre, Smart Manufacturing Research Institute, Universiti Teknologi MARA (UiTM) Cawangan Selangor, Kampus Puncak Alam, Puncak Alam 42300, Malaysia; 3 Department of Pharmacy, Hospital Kuala Lumpur, Kuala Lumpur 50586, Malaysia; 4 Fakultas Ilmu Kesehatan, Universitas Ubudiyah Indonesia, Kota Banda Aceh 23231, Indonesia; 5 School of Medical and Life Sciences, Sunway University, Sunway City 47500, Malaysia; 6 School of Pharmacy, KPJ Healthcare University College, Nilai 71800, Malaysia

**Keywords:** falls, fall-risk increasing drugs, older people, family caregivers, medication use

## Abstract

Background: A common contributory factor to falls is the use of medicines, especially those commonly known as “fall-risk increasing drugs” (FRIDs). The use of FRIDs is common among older people (OP). However, OP and their family caregivers (FCGs) are largely unaware of FRIDs and their risks in increasing the risk of falls (ROF). Methods: A booklet which aims to provide information on topics related to FRIDs was developed. The booklet was reviewed by a panel of 14 reviewers, and the content validity index (CVI) for each subsection of the booklet was computed. Pilot testing of the booklet utilized a pre-post intervention study design and included 50 OP and 50 FCGs as study participants. Perceived knowledge of the participants was assessed prior to and after completing the booklet. Participants’ opinions on the usefulness and usability of the booklet were also obtained. Results: The booklet contained eight sections and each subsection of the booklet had a CVI ranging from 0.93 to 1.00. Completing the booklet resulted in improved perceived knowledge scores for each perceived knowledge item among both the OP and FCG groups (all items: *p*-value < 0.001). The participants perceived the booklet as useful and usable, as evidenced by almost all the perceived usefulness and usability items having a score of over 4.0. Conclusions: The FRIDs booklet developed in this study had good content validity and was widely accepted by the OP and FCGs. The positive effect on the participants’ knowledge of topics related to FRIDs means that the booklet could be useful as a patient education tool to enhance FRIDs knowledge and awareness among OP and FCGs.

## 1. Introduction

The number of older people (OP) has escalated over recent decades [1]. By 2050, the global population of OP is expected to have doubled, and in 2100, it will have more than tripled [2]. In Malaysia, the proportion of OP in 2010 was only 5%, [3] but this has increased to 7.3% in 2022 [4]. The proportion is expected to increase to 14.5% in 2040 [3]. OP are generally associated with health problems, including chronic and complex multi-morbidities that may affect their quality of life. A major public health problem among OP globally is falls [5]. International studies have shown that around 30% of community-dwelling OP aged ≥ 65 years fall each year [6,7]. Alarmingly, OP who have experienced a fall in the previous year are more likely to fall again [8]. In Malaysia, studies conducted in nursing homes reported that 24.3% to 32.8% of OP residents had fallen [9,10,11]. Among Malaysian community-dwelling OP, previous studies have reported a fall prevalence ranging from 14.1% to 27.3% [10,12,13,14,15]. Falls can lead to injuries and mortality, making this issue a global public health concern [5,16].

Falls can occur due to multiple factors, such as poor strength, instability, visual impairment, and cognitive decline [17]. Thus, the measures to prevent falls in OP generally emphasize multifactorial assessment and intervention, including physical activity promotion, fall prevention education, home and environment modification, vitamin D supplementation, and visual assessment [18].

Many OP who fall tend to receive care and support for their activities of daily living from their family members [19,20,21]. Family caregivers (FCGs) should therefore have adequate knowledge about fall prevention strategies so that they can guide the OP under their care and prevent the re-occurrence of falls among them. Additionally, FCGs should partner with healthcare providers (HCPs) to decide and implement fall prevention strategies for the OP under their care [19].

A common contributory factor to falls is the use of medicines, especially those commonly known as “fall-risk increasing drugs” (FRIDs) [22]. Common FRIDs include antihypertensives, sedative-hypnotics, antipsychotics, antidepressants, and opioid analgesics [23,24,25,26,27,28]. These drugs increase the risk of falls (ROF) in OP mainly through their pharmacological actions, which can adversely affect the central nervous system or cause orthostatic hypotension (OSH) [29]. The use of FRIDs is common among OP, including those who are at ROF [30,31,32,33]. As reported in previous studies conducted in England, Spain, and Sweden, 65% to 93% of OP who required medical attention due to falls were using these drugs [30,31,32]. In a recent study conducted in Malaysia, the use of FRIDs was identified in 73.6% of OP living in thirteen residential aged care facilities. Additionally, 54.2% of FRIDs users in the study had a ROF, based on fall-risk assessment criteria [34].

Although the use of medicines has been regarded as a contributory factor to falls, many OP are unaware of this [35]. Bell et al. (2017) reported that the OP interviewed in their qualitative study did not regard the use of medicines as an important risk factor for falls, while none of them were familiar with FRIDs [36]. Poor awareness of FRIDs among OP was also noted in surveys conducted in Canada and the United States, with many OP in these studies being unaware that medications such as antihypertensives, anxiolytics, and hypnotics can increase the ROF in OP [37,38]. The level of FRID knowledge and awareness among FCGs has been under-investigated. However, in a study by Avila et al. (2015), almost 60% of FCGs participating in the study mentioned that they had no knowledge about fall prevention, while those who admitted being knowledgeable failed to mention any fall prevention strategies that target medication-related falls [39].

The low awareness of FRIDs among OP and FCGs was recognized by the European Geriatric Medicine Society Task and Finish group on FRIDs as an important issue to be addressed. This led to their recommendation that more effort was needed to disseminate FRIDs knowledge to OP [40]. Educating OP about FRIDs could empower them to become active players by sharing decision making with their HCPs to ensure they use their medications safely [40,41]. In addition, the increasing onus on FCGs to prevent falls among the OP under their care [42] means that the former should also be educated about FRIDs [40]. Unfortunately, previous studies have shown that the majority of OP had never received information from HCPs about the risk that their medications might increase their ROF [38,43]. In a focus group discussion (FGD), Malaysian HCPs (e.g., geriatricians, pharmacists, and nurses) directly involved in older patient care expressed a general opinion that there was limited HCP–patient communication about FRIDs, resulting in OP having limited awareness of the topic [44]. Additionally, previous studies have shown that many FCGs received no training and had limited access to information that could support them in managing the medications of the OP for whom they cared [45,46]. Hence, it is unsurprising that FCGs usually desire more information about the medications being used by the OP under their care [47,48,49].

The development of FRIDs education materials (EMs) to enhance the FRIDs awareness of both OP and FCGs has been recommended by HCPs and relevant professional bodies [40,44]. Such EMs can serve as patient education tools to facilitate FRIDs knowledge dissemination that can be referred to repeatedly by OP and which may empower them to communicate with HCPs about the safety of their medications [44]. Despite the many fall prevention EMs currently available, they generally emphasize fall prevention strategies such as nutrition education, home renovation, exercises, and managing the fear of falling [50,51,52]. Even where fall prevention EMs include information about medications, this does not adequately cover FRIDs information, such as side effects management and measures to ensure safe medication use [52,53]. Additionally, the existing EMs on fall prevention were generally designed only for care recipients (i.e., OP), not their FCGs.

The low awareness of FRIDs among OP and FCGs, the limited HCP-patient communication about FRIDs, and the limited coverage of information about FRIDs in the existing EMs on fall prevention indicated a core message: the need to improve efforts to educate OP about FRIDs. Thus, this study aimed to develop and pilot test a booklet on FRIDs as an initiative to improve the FRIDs knowledge of both OP and FCGs. The booklet was designed to provide knowledge and information on various topics related to FRIDs and thus serve as an EM that can be used as an educational strategy.

## 2. Methodology

**Figure 1:**
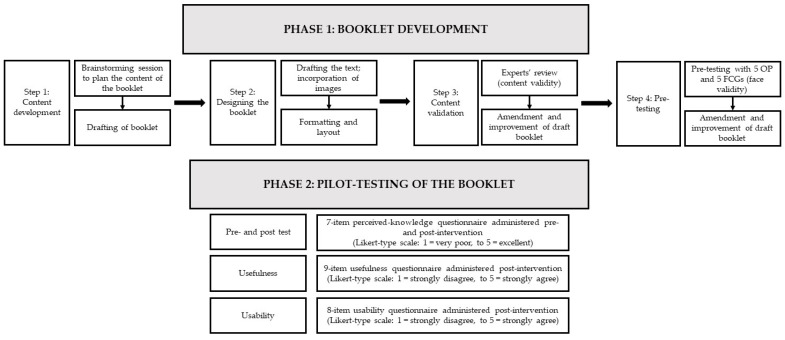
Schematic diagram of the methodological steps involved in the study (OP: older people; FCGs: family caregivers).

Figure 1 shows the schematic diagram of the methodological steps involved in this study. The study was conducted in two phases: (i) Phase 1: booklet development and (ii) Phase 2: pilot testing the booklet.

### 2.1. Phase 1: Booklet Development

#### 2.1.1. Step 1: Content Development

The booklet, which aims to provide information on topics related to FRIDs, is targeted at OP and their FCGs. Before the development of the booklet, a needs assessment was conducted that featured an FGD involving Malaysian geriatricians, nurses, and pharmacists. The study findings have been reported elsewhere [44], but they indicated the need for EMs on FRIDs due to the low awareness of FRIDs among OP. The HCPs in the FGD suggested that these EMs should ideally contain information on (i) FRIDs as a risk factor for falls; (ii) complications of falls; (iii) types of FRIDs; (iv) recognizing the side effects of FRIDs; (v) how these drugs increase the ROF; (vi) self-monitoring the drugs’ side effects; (vii) communication with HCPs about FRIDs; and (viii) the roles played by medication reviews in reducing the risks of FRIDs [44].

Additionally, a review of the existing EMs on fall prevention was conducted to identify any information gaps about FRIDs in those EMs [53]. The review revealed that the existing fall prevention EMs rarely contained information on medications as a risk factor for falls, certain types of FRIDs (e.g., antihyperglycemic agents and analgesics), certain side effects of FRIDs (e.g., OSH), the importance of medication reviews, or measures to ensure the safe use of FRIDs. Additionally, many of the reviewed EMs failed to advise that OP should communicate with HCPs about their medication use [53].

The findings from the FGD and review [44,53], as well as the information compiled from the relevant literature [18,26,27,29,40,54], were used as the foundation for a brainstorming session involving the research team members, two pharmacologists, and two pharmacists to plan a draft booklet. Consequently, two research team members, MSS and MSAW, drafted the initial version of the booklet. The booklet aimed to fulfill the following criteria: (i) address the recommendation from the previous FGD study regarding the ideal FRIDs-related content of EMs [44]; (ii) address the content gap regarding FRIDs in the existing EMs on fall prevention, as reported in the previous review [53]; (iii) boost the readers’ knowledge of topics related to FRIDs; and (iv) satisfy the readers in terms of the content and usability.

#### 2.1.2. Step 2: Designing the Booklet

The booklet was designed according to the guidelines for creating easy-to-understand EMs, which emphasize the appropriateness of the text, language, visuals, layout, and design, as well as the importance of pre-testing a newly developed EM [55,56]. The booklet was constructed using Malay, the official language of Malaysia. The booklet is in A5 size (210 mm × 148 mm) and has a total of 45 pages. The booklet was written in a conversational style since it had been suggested that this would present a more natural tone and be easier to understand [15]. The booklet was written in layman’s terms, so jargon, technical wording, and scientific language were avoided. This was to ensure that the booklet was readable and understandable by the target users. The Calibri font was used throughout the booklet, with 16 and 14 points for the sizes of the section and sub-section titles, and body text, respectively. The Calibri font and the sizes were selected after taking into account the types and sizes that would be suitable for OP [15]. Additionally, the booklet used an attractive layout and incorporated appropriate human and cartoon images to present a more casual tone.

#### 2.1.3. Step 3: Content Validation

The draft booklet was reviewed by a panel of 14 reviewers, consisting of pharmacy practice researchers (*n* = 6), hospital pharmacists (*n* = 3), community pharmacists (*n* = 3), and geriatricians (*n* = 2). The review was intended to assess the relevance of each subsection of the booklet, as well as evaluate the adequateness, essentiality, and clarity of the content. For each subsection of the booklet, the reviewers were requested to provide a rating from 1 to 4 (1 = not relevant; 2 = somewhat relevant; 3 = quite relevant; and 4 = very relevant) for the relevance of content. Consequently, the booklet’s content validity was determined by computing the content validity index (CVI) of each subsection. To calculate the CVI, the number of expert panelists providing ratings of “very relevant” (rating 4) and “quite relevant” (rating 3) for each subsection was divided by the total number of panelists (*n* = 14) [57].

Additionally, the reviewers were requested to provide a rating from 1 to 3 for the adequateness (1 = inadequate; 2 = quite adequate; and 3 = adequate); essentiality (1 = not essential; 2 = useful but not essential; and 3 = essential); and clarity (1 = not clear; 2 = item needs some revision; and 3 = very clear) of each subsection of the booklet. For the adequateness, essentiality, and clarity, the average scores were calculated, with the maximum possible scores of 3.0.

Then, using a short questionnaire, the reviewers were requested to provide feedback on the suitability of several aspects of the booklet (e.g., the title, images, and fonts). Lastly, they were requested to comment on how the wording, layout, and formatting of the booklet could be improved.

#### 2.1.4. Step 4: Pre-Testing

The booklet was pre-tested by ten individuals (OP, *n* = 5; and FCGs, *n* = 5). The purpose of the pre-testing was to ensure that the booklet had face validity and was suitable for the target users (i.e., OP and FCGs). The pre-testing participants were recruited from the researchers’ network. The participants were requested to read and review the booklet. After they had done so, one-to-one interviews were held in which one research team member (MSS) requested that the participants highlight any parts or sentences that were difficult to understand or any images they considered irrelevant. They were also asked to comment on the suitability of the fonts and layout employed. Revisions were made based on the participants’ feedback.

### 2.2. Phase 2: Pilot Testing of the Booklet

#### 2.2.1. Study Design and Setting

The study design was a pre–post intervention in which each participant acted as his or her own control. The participants were conveniently sampled from five districts in Kedah, a state in the north of Malaysia. Data collection was performed in May and June 2022.

#### 2.2.2. Sample Population

The study recruited OP and FCGs as the study participants. The participant selection was performed using convenience sampling. Potential participants were approached at public places, such as supermarkets, malls, and places of worship. OP could be admitted into the study if they fulfilled the following criteria: they (i) were 60 years of age or older; (ii) were using at least one of the following: antihypertensives, heart disease medications, hypnotics, psychotropics, opioid analgesics, antihyperglycemics, or insulin; (iii) could understand and read Malay; and (iv) agreed to participate. The inclusion criteria for the FCGs were: (i) individuals aged 18 years or older; (ii) able to understand and read Malay; and (iii) taking care of an older person aged 60 years or more who was using at least one type of medication listed in the criteria for the OP group. 

#### 2.2.3. Study Instrument

A questionnaire to assess the extent of the participants’ perceived knowledge of topics related to FRIDs (Table A1) was developed based on the booklet content. The questionnaire consisted of seven questions for which the answer options utilized a Likert-type scale ranging from 1 = very poor to 5 = excellent. Additionally, two sets of questionnaires were developed to assess the participants’ perceptions of the usefulness (nine items) and usability (eight items) of the booklet (Table A2 and Table A3). In this study, the pre-existing general questionnaire to measure the usefulness and usability of a product (i.e., the booklet) was not utilized since the study specifically aimed to seek participants’ perceptions of the usefulness and usability of the booklet content. The usefulness questionnaire concerned the extent of the participants’ satisfaction with how well the booklet provided information about FRIDs topics. On the other hand, the usability questionnaire sought the participants’ opinions on the appropriateness of various technical aspects of the booklet (e.g., the font, images, and time to complete reading). For these two questionnaires, the answer options included a Likert-type scale ranging from 1 = strongly disagree to 5 = strongly agree.

All three questionnaires were reviewed by six public health research experts to assess the relevance of each questionnaire item. The reviewers provided ratings from 1 = not relevant to 4 = very relevant for each item. The reviewers were also requested to comment on the clarity of the items. Their evaluation of the questionnaire items revealed that all the items were relevant and acceptable, with CVI scores ranging from 0.83–1.0 [57]. Additionally, the reviewers commented on how the wording of some items could be improved. Based on the reviewers’ feedback, minor amendments were made to enhance the clarity of the questionnaire items.

#### 2.2.4. Study Procedure

The participants who agreed and consented to participate in the study were first requested to respond to the questions in the perceived-knowledge questionnaire. Consequently, they were provided with the booklet and requested to read the content, guided by a research team member (MSS) who assisted them in navigating through the booklet. Reading the booklet took approximately 20 to 30 min.

Having read the booklet, the perceived-knowledge questionnaire was again administered to the participants. They were also requested to answer the questions in the usefulness and usability questionnaires. The responses were collected from the participants using the interviewer-administered questionnaire method. All the participants were offered anonymity and confidentiality. Each was provided with an incentive of MYR 50 (~USD 11) to participate in the study.

#### 2.2.5. Ethical Approval

The study received ethical approval from the Research Ethics Committee of *Universiti Teknologi* MARA (REC/05/2022 [PG/FB/9]).

#### 2.2.6. Statistical Analysis

The demographic information of the participants was analyzed using descriptive statistics. The distribution of the participants’ responses for each item in the perceived-knowledge questionnaire is presented using frequency and percentages. The Chi-squared or Fisher’s exact test was used to determine the difference in the percentages of the participants’ responses. To assess the changes in the participants’ perceived knowledge, the pre- and post-test survey median scores were compared using the Wilcoxon signed-rank test, as the data were not normally distributed. For the usefulness and usability items, the mean score (M) and standard deviation (SD) values were computed and presented.

## 3. Results

### 3.1. Phase 1: Booklet Development

#### 3.1.1. Content Development

The draft booklet is entitled “*Penggunaan Ubat dan Risiko Jatuh: Maklumat untuk Warga Emas dan Penjaga*” [Medication Use and Risks of Falls: Information for Older People and Caregivers] and it contained eight sections, as shown in Table 1. Sample pages of the booklet are provided in Table A4.

#### 3.1.2. Designing the Booklet

The booklet content was organized as listed in Table 1. Each page was designed with consideration of suitable text, images, and layout to ensure that the booklet would be not only informative but also attractive. The draft booklet was revised several times before it was submitted for content validation by the expert panel.

#### 3.1.3. Content Validation

Based on Lynn (1986), with over nine expert individuals, a CVI score of at least 0.78 for each content item would be considered acceptable [58]. The CVI calculation showed that the relevance of each subsection of the booklet was acceptable, since the CVI values ranged from 0.93 to 1.00 (Table 2). Furthermore, the mean adequateness, essentiality, and clarity scores of the subsections were also acceptable, with mean scores ≥ 2.5. Table 3 presents the reviewers’ opinions on the suitability of the booklet title, images, fonts, and design, which received high levels of agreement from the reviewers. The reviewers also provided feedback on the wording, layout, and formatting of the booklet. Minor amendments were made based on these comments. 

#### 3.1.4. Pre-Testing

The pre-testing of the booklet showed that it was satisfactory in terms of the content, format, and layout. All ten participants in the pre-testing study indicated they understood the information in the booklet. The images used were perceived as appropriate by the participants.

### 3.2. Phase 2: Pilot Testing the Booklet

#### Demographic Characteristics

Table 4 shows the demographic characteristics of the participants. In total, 50 OP and 50 FCGs were recruited for the study. Of the OP group, 56% (28/50) were female and most were in the 60 to 69 age group (80%, 40/50); the remainder (20%, 10/50) were 70 years old or older. The majority (70%, 35/50) were using ≥4 medications daily. At the time of the study, the majority of the OP participants were using antidiabetics (86%, 43/50) or antihypertensives (80%, 40/50). Within this group, the prevalence of a fall in the past year was 6% (3/50).

Meanwhile, an equal number of female (*n* = 25) and male (*n* = 25) FCGs were recruited. Most were 40 years old or older (64%, 32/50). The majority indicated that the OP under their care were taking ≥4 medications daily (76%, 38/50), with most using antidiabetics (84%, 42/50) or antihypertensives (72%, 36/50). In total, three FCGs (6%) stated that the OP under their care had fallen in the past year. All the participants from the OP and FCG groups were Malays. 

### 3.3. Comparison of Respondents’ Perceived Knowledge before and after the Intervention within Groups

Table 5 shows the comparison of the participants’ perceived knowledge before and after the intervention within groups. Prior to the intervention, the OP appeared to have low perceived knowledge about the side effects of medications that could increase the ROF, measures to overcome such side effects, and medication reviews (all three topics: median = 1.0, IQR = 2.0, range = 1.0–4.0). Among the FCGs, low perceived knowledge was noted in regard to measures to overcome the side effects of medications that could increase the ROF and medication reviews (both topics: median = 1.0, IQR = 2.0, range = 1.0–4.0).

Before the intervention, the distribution of the participants’ responses showed that the majority of the OP rated as very poor or poor their perceived knowledge of measures to overcome the side effects of FRIDs (66%, 33/50) and medication reviews (72%, 36/50). Additionally, about 60% (28/50) of them perceived their knowledge about the side effects of FRIDs as very poor or poor. Half of them rated their perceived knowledge of the negative implications of falls (50%, 25/50) and the risks of falls (46%, 23/50) as very poor or poor.

Two-thirds of the FCGs rated their perceived knowledge of measures to overcome the side effects of FRIDs (66%, 33/50) and medication reviews (68%, 34/50) as very poor or poor. Prior to the intervention, the Chi-squared test showed that the percentage of OP rating their perceived knowledge of the negative implications of falls and the side effects of FRIDs as poor or very poor was significantly higher than the percentage of FCGs who did so. On the other hand, a significantly higher percentage of FCGs rated their perceived knowledge of the safe use of medicines as very poor or poor.

Completing the booklet resulted in improved perceived knowledge scores for each perceived knowledge item among both the OP and FCG groups (all items: *p*-value < 0.001) (Table 5). Post-intervention, none of the participants rated their perceived knowledge as very poor or poor for each of the booklet topics. The exception was one OP participant who provided a poor rating for his perceived knowledge of medication reviews. Post intervention, the Chi-squared test showed that compared to the OP, a significantly higher percentage of FCGs rated as good to excellent their perceived knowledge of the negative implications of falls, the risks of falls, and medication reviews.

### 3.4. Perceived Usefulness and Usability of the Booklet

In general, the participants perceived the booklet as useful and usable, as evidenced by almost all the perceived usefulness (Table 6) and usability (Table 7) items having a score of over 4.0. In terms of the booklet’s usefulness, both the OP and FCGs strongly agreed that it provided clear guidance on how to communicate with HCPs about medications (OP: M = 4.68, SD = 0.47; and FCGs: M = 4.78, SD = 0.42). Additionally, the OP strongly agreed that the booklet did not induce fear of taking medications (M = 4.68, SD = 0.47) and that the booklet was useful (M = 4.68, SD = 0.55). In comparison, the mean score for the item “*The content of the booklet does not induce fear of taking medications*” was slightly lower than 4.0 among the FCGs (M = 3.96, SD = 0.83).

In terms of the booklet’s usability, the OP provided the lowest ratings for the appropriateness of the images it contained (M = 4.36, SD = 0.60). On the other hand, the FCGs provided high ratings for that aspect (M = 4.62, SD = 0.57). Both OP and FCGs strongly agreed that the font used in the booklet was suitable (OP: M = 4.76, SD = 0.43; and FCGs: M = 4.74, SD = 0.44).

## 4. Discussion

To our knowledge, this is the first study to describe the development and pilot testing of a booklet focusing on medications that can increase the ROF. The statistically significant increase in the perceived knowledge of the participants about various topics contained in the booklet provides preliminary evidence of the booklet’s success. Additionally, the participants were satisfied with its content and technical aspects.

The booklet’s strengths are founded in the comprehensive development of the EM, which was based on inputs from previous studies [44,53], EM development guidelines [15,56], and the booklet’s content validation. The booklet addressed gaps identified in a previous FGD regarding EMs on FRIDs [44] and a review of fall prevention EMs [53]. The expert evaluation of the booklet provided further evidence that relevant, valid, and accurate information about FRIDs were included in the content. Each subsection of the booklet received excellent individual CVI scores and was regarded as adequate, essential, and clear. 

Before reading the booklet, more than half of the OP in this study reported poor knowledge about the side effects of FRIDs. This was consistent with the findings of Wien et al. (2006), who showed that the majority of OP surveyed in their study were unaware that the side effects of common FRIDs (e.g., hypnotics, anxiolytics, and antihypertensives) could increase the ROF [37]. Similarly, Leonetti and Lee (2014) reported that the majority of OP in their survey did not recognize hypnotics, psychotropics (e.g., antidepressants and anxiolytics), anticholinergics, or orthostasis-causing drugs as FRIDs [38]. In this study, FCGs also appeared to know little about the side effects of FRIDs, with about 40% indicating poor knowledge about the topic. We also noted that the majority of OP and FCGs in this study gave poor ratings for their knowledge about measures to overcome the side effects of FRIDs. Loke et al. (2018) also reported that more than half of the OP they surveyed were unaware of measures to reduce medication-related falls [43]. Other topics for which many OP and FCGs rated their knowledge as poor included fall risks and medication reviews.

The current findings indicate there is a compelling reason to provide education to OP and FCGs about FRIDs, given that they demonstrated poor knowledge about such topics. In the study by Leonetti and Lee (2014), OP who received patient education about FRIDs demonstrated significantly greater awareness of the topic, thus showing the value of patient education about FRIDs for OP [38]. The present study further supported the view that patient education plays a role in boosting OP and FCGs’ knowledge of FRIDs. In this study, the median perceived knowledge score for each topic in the booklet increased significantly after the booklet had been read completely.

Falls management guidelines recommend changes to, or the withdrawal of, medications that are contributing to falls and a loss of balance [18]. Such medications can be identified through medication reviews by HCPs, especially pharmacists. Such reviews enable HCPs to assess the appropriateness of OPs’ medication use and resolve any drug-related problems (e.g., medication duplication, adverse drug reactions, or drug–drug interactions) [59]. Additionally, they can evaluate whether an FRID should be discontinued or the dose reduced to lessen the ROF in OP [60,61]. Of note, a previous trial demonstrated the statistically significant falls-reduction benefit of deprescribing psychotropics [62].

However, in this study, a high proportion of OP and FCGs indicated they had poor knowledge about the role of medication reviews in reducing the ROF. This finding supported the result obtained in a previous FGD, in which HCPs agreed that OP were generally unaware of the role of, and need for, medication reviews [44]. An integrative review by Wilkinson et al. (2018) also suggested that medication reviews were underutilized by OP and FCGs as a fall prevention strategy [19]. Therefore, it was a reassuring finding that the booklet improved the participants’ knowledge about medication reviews. An awareness and knowledge of medication reviews can potentially empower OP to have their medications reviewed by HCPs or encourage FCGs to ensure the OP under their care obtain this service.

Hawe et al. (1990) recommended investigating the acceptance of EM by the target users [63]. In the current study, this was performed through the usefulness and usability questionnaires, which sought the participants’ opinions on how well the booklet provides information about FRIDs, as well as the suitability of various aspects, such as the content, design, and images. The findings from our study show that both the OP and FCG participants were generally satisfied with the booklet’s content and technical aspects. The booklet’s acceptance among the study participants can be attributed to the thorough development and design of the EM, which were based on guidelines [15,56] and comprehensive feedback from experts in the field.

In a previous a study, HCPs expressed concerns about EMs related to FRIDs, suggesting they may induce fear among OP about taking medications because the OP had been exposed to knowledge of the potential risks [44]. This could potentially result in OP rejecting their prescribed medications [64]. In this study, the booklet emphasized the need for information on not only the potential risks of FRIDs but also the reasons for which drugs had been prescribed and the importance of taking them. The booklet also incorporated information on recognizing the side effects of FRIDs and measures to overcome them. The booklet pilot testing results demonstrated that the participants, particularly the OP, generally agreed that the booklet did not cause fear of taking medications. However, it was noted that the FCGs expressed less agreement than the OP regarding this issue. This may have been because the FCGs were concerned about the occurrence of side effects among the OP under their care and the possible risks to which they were exposed. The findings suggest the need for FCG education reinforcing the facts that the potential risks of FRIDs can be avoided or minimized and that these drugs can be used safely. This could be further improved in the booklet or be provided verbally by HCPs.

In this study, the booklet was developed in print form. The strengths of this type of EM included people’s perceptions that this was useful and that it could serve as a continuous source of information by conveying basic and repetitive information [65]. Additionally, having the booklet in print form could be a valuable patient education tool during consultation with HCPs. For future use, the booklet could be transformed into a flipchart or infographics, as these formats have been shown to be potentially useful for patient education [66,67,68]. In addition, due to the growing interest in online platforms as a means for the public to obtain health-related information [69], the booklet could be converted into e-material and distributed through the Internet and on social media.

### Limitations of the Study

The current study has several limitations. First, the booklet developed in this study mainly emphasizes FRIDs. It does not contain other fall prevention measures, such as exercise or home modifications [70]. However, the existing fall prevention EMs generally emphasize fall prevention strategies by targeting behavioral and environmental fall risk factors [52], but they place little emphasis on medications and their role in increasing the ROF [52,53]. Thus, using the booklet developed in this study could complement the existing fall prevention EMs. Another limitation of the booklet is the inclusion of only selected types of FRIDs in the content (i.e., antihypertensives, heart disease medications, hypnotics, psychotropics, opioid analgesics, antihyperglycemic agents, and insulin). Other drugs, such as antihistamines and anticholinergic medications, which may increase the ROF in OP due to their anticholinergic side effects, were not included in the booklet. Future researchers may consider updating the booklet with such information.

Additionally, the booklet was developed in Malay. All the participants in the study were Malays and proficient in that language. Considering that Malaysia is a multi-national country and that many citizens use Chinese, Tamil, or English as their main spoken language, the booklet should be translated into other languages. Furthermore, the booklet developed and tested in this study was designed for OP and FCGs who could read. The booklet may be of limited use for illiterate OP or FCGs, or those with cognitive and visual impairments. Furthermore, the authors recruited OP who were using at least one type of medication that was known to increase the ROF, as well as FCGs who were caring for OP who had been prescribed such medications. The level of falls risk among the OP was not assessed before their inclusion in the study. The inclusion of participants with a low falls risk could have influenced the responses to the questionnaires. Future studies could include OP belonging to different falls risk categories so that different responses to the booklet can be obtained and analyzed.

To measure the effects of the booklet, a pre–post intervention study design was utilized with a sample of OP and FCGs who had been recruited using convenience sampling. The study design was limited due to the lack of randomized participant recruitment, making the results more prone to bias. Future studies may consider randomly selecting a group of study participants and a group of controls, with the pre- and post-test being administered to both groups over the same time interval but with only the study group receiving the booklet [71]. Moreover, the pre- and post-test survey collected the participants’ perceived knowledge about the topics covered in the booklet rather than their actual knowledge. Future researchers could develop a knowledge assessment tool to better measure knowledge changes as a result of the booklet. Additionally, only a small number of participants were included in the pilot testing of the booklet. Although this number of participants was appropriate for the pilot study [72], more participants could be recruited if the booklet is tested again in the future.

## 5. Conclusions

The FRIDs booklet developed in this study had good content validity and was widely accepted by the OP and FCGs. Reading the booklet in full resulted in statistically significantly greater perceived FRIDs knowledge among the OP and FCGs. This demonstrated that the booklet had a positive effect on the participants’ knowledge of topics related to FRIDs by narrowing many of the identified gaps, as assessed by a pretest survey completed before they read the booklet. Thus, the booklet could be useful as a patient education tool to enhance FRIDs knowledge and awareness among OP and FCGs. Future studies could consider developing the EM in other common Malaysian languages (e.g., English, Mandarin, and Tamil) to meet the demand of the multiracial OP in Malaysia, while the EM could also be tested among a larger sample size of OP.

## Figures and Tables

**Table 1:** Content of the draft booklet.

Section	Content
Introduction	Descriptions of the purposes of the booklet; a short overview of the prevalence of falls among OP in Malaysia and the complications of falls.
Fall risks	Includes the fall risk assessment criteria, allowing readers to recognize scenarios that would increase the ROF in OP.
Types of medications associated with falls	This section explains medication as a risk factor for falls. It is further divided into several subsections: (i) antihypertensives and heart disease medications; (ii) hypnotics and psychotropics; (iii) opioid analgesics; and (iv) antihyperglycemic agents and insulin. Each subsection contains information on the reasons for the prescribing of the medications; the importance of taking the medications as prescribed; examples of medications in each drug class; and how the side effects of the medications can increase the ROF.
Side effects of medications that can increase the ROF and the management of these side effects	This section is divided into three subsections: (i) OSH; (ii) sleepiness and drowsiness; and (iii) hypoglycemia. Each subsection reiterates the types of medications that can cause the side effects. Additionally, each subsection outlines the signs and symptoms of the side effects; how the side effects increase the ROF; the recommended actions if the side effects occur; and measures to minimize the ROF due to these side effects.
Communication with HCPs about medications	This section outlines the aspects of medications that must be discussed with HCPs (e.g., the purposes and outcomes of medications, as well as their potential interactions with other medications).
Medication review	This section describes medication review; its purposes; reasons why OP should have their medications reviewed; what should be reviewed; and how to obtain medication review services from HCPs.
Safe use of medicines	This section provides recommendations to ensure the safe use of medicines (e.g., taking medications as directed, not sharing medications with others, and not using any medications without consulting with HCPs).
Conclusion	The conclusion summarizes the important points regarding falls and FRIDs.

OP: older people; ROF: risk of falls; OSH: orthostatic hypotension; HCPs: healthcare providers.

**Table 2:** Relevance, adequateness, essentiality, and clarity of the content of each subsection of the booklet.

Section/Subsection	Relevance ^a^	Adequateness ^b^	Essentiality ^c^	Clarity ^d^
	CVI	Mean		
**Section 1: Introduction**				
Introduction	1.00	2.86	2.93	2.79
Purposes of booklet	1.00	3.00	3.00	2.86
Overview of the prevalence of falls among OP in Malaysia and complications of falls	1.00	2.93	2.93	2.93
**Section 2: Fall risks**				
Fall risks	0.93	2.93	3.00	2.57
**Section 3: Types** **of medications associated with falls**				
Medications and risk of falls	1.00	2.93	2.93	3.00
Antihypertensives and heart disease medications	1.00	2.93	2.86	2.50
Hypnotics and psychotropics	1.00	2.79	2.86	2.64
Opioid analgesics	1.00	3.00	3.00	2.86
Antihyperglycemic agents and insulin	1.00	3.00	3.00	2.71
**Section 4: Side** **effects of medications that can increase the ROF and the management of these side effects**				
OSH	1.00	2.79	2.86	2.64
Sleepiness and dizziness	1.00	3.00	2.86	2.79
Hypoglycemia	1.00	2.86	2.86	2.79
**Section 5: Communication** **with HCPs about medications**				
Medication information	0.93	2.93	2.93	2.79
Medication intake and storage	0.93	2.93	2.93	2.93
Outcomes of taking medications	0.93	2.93	2.93	2.93
Potential interactions between medications and food	0.93	2.86	2.93	2.86
Potential risks that medications would increase the ROF	0.93	2.93	2.93	2.93
**Section 6: Medication** **review**				
Medication review	0.93	2.71	2.71	2.57
**Section 7: Safe** **use of medicines**				
Safe use of medicines	0.93	2.86	2.86	2.64
**Section 8: Conclusion**				
Conclusion	1.00	2.93	2.93	2.93

CVI: content validity index; OP: older people; ROF: risk of falls; OSH: orthostatic hypotension. ^a^ Relevance rating: 1 = not relevant; 2 = somewhat relevant; 3 = quite relevant; 4 = very relevant. ^b^ Adequateness rating: 1 = inadequate; 2 = quite adequate; 3 = adequate. ^c^ Essentiality rating: 1 = not essential; 2 = useful but not essential; 3 = essential. ^d^ Clarity rating: 1 = not clear; 2 = item needs some revision; 3 = very clear.

**Table 3:** Reviewers’ feedback results (*n* = 14).

Aspect	% Agree
The title of the booklet is suitable	93
The images used in the booklet are appropriate	93
The font used is suitable	100
The font sizes are suitable	100
The booklet formatting is appropriate	100

**Table 4:** Demographic characteristics of OP (*n* = 50) and FCGs (*n* = 50) who participated in the study.

Demographic Characteristics		*n* (%)
**OP Participants**		
Gender	Male Female	22 (44) 28 (56)
Age	60–69 years old ≥70 years old	40 (80) 10 (20)
Race	Malay	50 (100)
Number of medications	<4 medications ≥4 medications	15 (30) 35 (70)
Use of medications associated with falls ^a^	Antidiabetics Antihypertensives Opioid analgesics Psychotropics or hypnotics	43 (86) 40 (80) 5 (10) 3 (6)
History of falls in the past 12 months	Yes No	3 (6) 47 (94)
**FCG participants**		
Gender	Male Female	25 (50) 25 (50)
Age	<40 years old ≥40 years old	18 (36) 32 (64)
Race	Malay	50 (100)
Number of medications used by OP under care	<4 medications ≥4 medications	12 (24) 38 (76)
Use of medications associated with falls by OP under care ^a^	Antidiabetics Antihypertensives Opioid analgesics Psychotropics or hypnotics	42 (84) 36 (72) 7 (14) 3 (6)
History of falls in the past 12 months among OP under care	Yes No	3 (6) 47 (94)

OP: older people; FCG: family caregiver. ^a^ Participants could provide more than one response, so the response percentages do not add up to 100%.

**Table 5:** Comparison of participants’ perceived knowledge before and after the intervention within and between groups (OP, *n* = 50 and FCGs, *n* = 50).

Topic	Group	Pre-Test			*p* Value ^a^	Post-Test			*p* Value ^a^	Median Pre-Test Score (Interquartile Range), Min–Max	Median Post-Test Score (Interquartile Range), Min–Max	*p* Value (within Group) ^b^
		Very Poor and Poor	Neither Poor nor Good	Good—Excellent		Very Poor and Poor	Neither Poor nor Good	Good—Excellent				
		*n* (%)				*n* (%)						
Negative implications of falls in OP	OP	25 (50)	16 (32)	9 (18)	<0.001	0 (0)	10 (20)	40 (80)	0.014	2.5 (1.0), 1.0–5.0	4.0 (1.0), 3.0–5.0	<0.001
	FCGs	6 (12)	27 (54)	17 (34)		0 (0)	2 (4)	48 (96)		3.0 (1.0), 1.0–5.0	5.0 (1.0), 3.0–5.0	<0.001
Risks of falls	OP	23 (46)	22 (44)	5 (10)	0.571	0 (0)	27 (54)	23 (46)	0.001	3.0 (1.0), 1.0–4.0	3.0 (1.0), 3.0–5.0	<0.001
	FCGs	18 (36)	27 (54)	5 (10)		0 (0)	11 (22)	39 (78)		3.0 (2.0), 1.0–5.0	4.0 (1.0), 3.0–5.0	<0.001
Side effects of medications that can increase the risk of falls in OP	OP	28 (56)	19 (38)	3 (6)	0.042	0 (0)	2 (4)	48 (96)	1.000 ^c^	1.0 (2.0), 1.0–4.0	4.0 (1.0), 3.0–5.0	<0.001
	FCGs	19 (38)	20 (40)	11 (22)		0 (0)	1 (2)	49 (98)		3.0 (2.0), 1.0–5.0	5.0 (1.0), 3.0–5.0	<0.001
Measures to overcome the side effects of medications that can increase the risk of falls in OP	OP	33 (66)	16 (32)	1 (2)	0.833	0 (0)	1 (2)	49 (98)	0.204 ^c^	1.0 (2.0), 1.0–4.0	4.0 (1.0), 3.0–5.0	<0.001
	FCGs	33 (66)	15 (30)	2 (4)		0 (0)	5 (10)	45 (90)		1.0 (2.0), 1.0–4.0	4.0 (1.0), 3.0–5.0	<0.001
Topics of discussion with HCPs about medications	OP	10 (20)	18 (36)	22 (44)	0.054	0 (0)	0 (0)	50 (100)	-	3.0 (1.0), 1.0–5.0	5.0 (1.0), 3.0–5.0	<0.001
	FCGs	7 (14)	30 (60)	13 (26)		0 (0)	0 (0)	50 (100)		3.0 (1.0), 1.0–4.0	5.0 (1.0), 3.0–5.0	<0.001
Medication reviews	OP	36 (72)	13 (26)	1 (2)	0.808	1 (2)	9 (18)	40 (80)	0.045	1.0 (2.0), 1.0–4.0	4.0 (1.0), 2.0–5.0	<0.001
	FCGs	34 (68)	14 (28)	2 (4)		0 (0)	2 (4)	48 (96)		1.0 (2.0), 1.0–4.0	5.0 (1.0), 3.0–5.0	<0.001
Safe use of medicines	OP	10 (20)	27 (54)	13 (26)	0.029	0 (0)	5 (10)	45 (90)	0.204 ^c^	3.0 (1.0), 1.0–5.0	5.0 (1.0), 3.0–5.0	<0.001
	FCGs	14 (28)	14 (28)	22 (44)		0 (0)	1 (2)	49 (98)		3.0 (2.0), 1.0–5.0	5.0 (1.0), 3.0–5.0	<0.001

OP: older people; FCGs: family caregivers. ^a^ Chi-squared test used unless stated otherwise. ^b^ Wilcoxon signed-rank test used. ^c^ Fisher’s exact test used.

**Table 6:** Perceptions of the participants about the booklet’s usefulness (OP, *n* = 50 and FCGs, *n* = 50).

Item	OP (*n* = 50)	FCGs (*n* = 50)	All (*n* = 100)
The booklet clearly explains the consequences of falls in OP	4.60 ± 0.54	4.44 ± 0.50	4.52 ± 0.52
2. The booklet clearly explains the side effects of medications that can increase the risk of falls in OP	4.30 ± 0.68	4.46 ± 0.61	4.38 ± 0.65
3. The booklet clearly explains the preventative measures for falls due to the side effects of medications	4.40 ± 0.61	4.42 ± 0.61	4.41 ± 0.61
4. The booklet provides clear guidance on how to communicate with HCPs about the medication taken	4.68 ± 0.47	4.78 ± 0.42	4.73 ± 0.45
5. The booklet clearly explains the services offered by HCPs to review medications and ensure they are safe	4.20 ± 0.67	4.32 ± 0.65	4.26 ± 0.66
6. The booklet offers clear guidance on the safe use of medicines	4.42 ± 0.64	4.46 ± 0.58	4.44 ± 0.61
7. The booklet clearly explains the importance of medications and their side effects	4.34 ± 0.59	4.30 ± 0.65	4.32 ± 0.62
8. The booklet content does not induce fear of taking medications	4.68 ± 0.47	3.96 ± 0.83	4.32 ± 0.76
9. The booklet content on medications and falls risk is useful	4.68 ± 0.55	4.44 ± 0.58	4.56 ± 0.57

OP: older people; FCGs: family caregivers.

**Table 7:** Perceptions of the participants about the booklet’s usability (OP, *n* = 50 and FCGs, *n* = 50).

Items	OP (*n* = 50)	FCGs (*n* = 50)	All (*n* = 100)
The booklet content is easy to understand	4.42 ± 0.58	4.52 ± 0.58	4.47 ± 0.58
2. The terminology used in the booklet is easy to understand	4.44 ± 0.54	4.42 ± 0.58	4.43 ± 0.56
3. The sentences used in the booklet are easy to understand	4.64 ± 0.53	4.46 ± 0.71	4.55 ± 0.63
4. The images in the booklet are appropriate	4.36 ± 0.60	4.62 ± 0.57	4.49 ± 0.60
5. The type of font used in the booklet is suitable	4.76 ± 0.43	4.74 ± 0.44	4.75 ± 0.44
6. The font sizes are easy to read	4.78 ± 0.42	4.64 ± 0.49	4.71 ± 0.46
7. The booklet is suitable to be read by OP	4.66 ± 0.56	4.46 ± 0.73	4.56 ± 0.66
8. The time taken to finish reading the booklet is suitable	4.42 ± 0.64	4.48 ± 0.68	4.45 ± 0.66

OP: older people; FCGs: family caregivers.

## Data Availability

The data presented in this study are available on request from the corresponding author.

## Abbreviations

OP: Older people; FCGs: Family caregivers; HCPs: Healthcare providers; FRIDs: Fall-risk increasing drugs; ROF: Risk of falls; OSH: Orthostatic hypotension; FGD: focus group discussion; EMs: Education materials; CVI: Content validity index; M: Mean score; SD: Standard deviation.

## Appendix A

**Table A1:** Perceived-knowledge questionnaire ^a^.

Item
Please rate the extent of your knowledge about:
the negative implications of falls in older people.
2. your risks of falls.
3. the side effects of medications that can increase the risk of falls in older people.
4. the measures to overcome the side effects of medications that can increase the risk of falls in older people.
5. the topics to discuss with healthcare providers about medications.
6. medication reviews.
7. the safe use of medicines.

^a^ The questionnaire used a Likert-type scale ranging from 1 = very poor to 5 = excellent for the answer options.

**Table A2:** Usefulness questionnaire ^a^.

Item
The booklet clearly explains the consequences of falls in older people.
2. The booklet clearly explains the side effects of medications that can increase the risk of falls in older people.
3. The booklet clearly explains the preventative measures for falls due to the side effects of medications.
4. The booklet provides clear guidance on how to communicate with healthcare providers about the medication taken.
5. The booklet clearly explains the services offered by healthcare providers to review medications and ensure they are safe.
6. The booklet offers clear guidance on the safe use of medicines.
7. The booklet clearly explains the importance of medications and their side effects.
8. The booklet content does not induce fear of taking medications.
9. The booklet content on medications and falls risk is useful.

^a^ The questionnaire used a Likert-type scale ranging from 1 = strongly disagree to 5 = strongly agree for the answer options.

**Table A3:** Usability questionnaire ^a^.

Item
The booklet content is easy to understand.
2. The terminology used in the booklet is easy to understand.
3. The sentences used in the booklet are easy to understand.
4. The images in the booklet are appropriate.
5. The type of font used in the booklet is suitable.
6. The font sizes are easy to read.
7. The booklet is suitable to be read by older people.
8. The time taken to finish reading the booklet is suitable.

^a^ The questionnaire used a Likert-type scale ranging from 1 = strongly disagree to 5 = strongly agree for the answer options.

**Table A4:** Sample pages of the booklet.

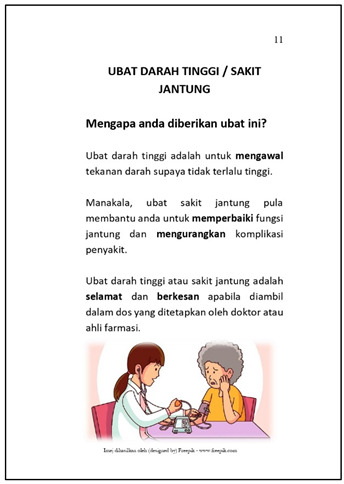
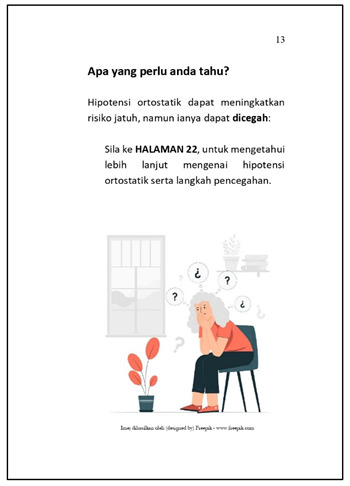

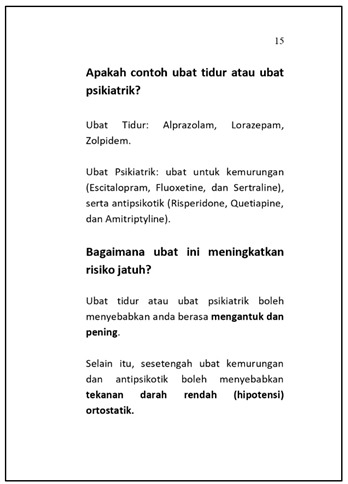
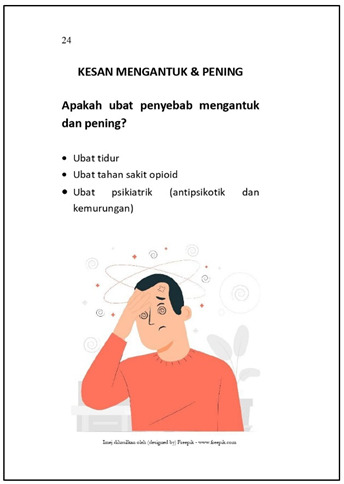

	
	
Images were designed by Freepik (www.freepik.com (accessed on 1 June 2022))	
